# Stent-assisted WEB embolization: aneurysm characteristics, outcome and case report of a WEB delivered through a stent

**DOI:** 10.1007/s00701-022-05115-y

**Published:** 2022-01-17

**Authors:** Lukas Goertz, Thomas Liebig, Eberhard Siebert, Muriel Pflaeging, Robert Forbrig, Lenhard Pennig, Erkan Celik, Nuran Abdullayev, Marc Schlamann, Franziska Dorn, Christoph Kabbasch

**Affiliations:** 1 Faculty of Medicine and University Hospital, Department of Radiology and Neuroradiology, University of Cologne, Kerpener Strasse 62, 50937 Cologne, Germany; 2 Department of Neuroradiology, University Hospital Munich (LMU), Munich, Germany; 3 Department of Neuroradiology, University Hospital of Berlin (Charité), Berlin, Germany; 4 Faculty of Medicine and University Hospital, Center for Neurosurgery, University of Cologne, Cologne, Germany; 5 Department of Neuroradiology, University Hospital Bonn, Bonn, Germany

**Keywords:** Endovascular, Intracranial aneurysm, Morbidity, Woven Endobridge

## Abstract

**Purpose:**

Woven Endobridge (WEB) embolization has become a well-established endovascular treatment option for wide-necked bifurcation aneurysms. The objective was to analyse cases that required additional stent-implantation.

**Methods:**

Images of 178 aneurysms ≤ 11 mm treated by WEB only or by WEB plus stent were retrospectively reviewed, evaluating aneurysm characteristics, procedural specifics, adverse events and angiographic results. Moreover, we report a case of a WEB delivered through a previously implanted stent.

**Results:**

Additional stent implantation was performed in 15 patients (8.4%). Baseline patient and aneurysm characteristics were comparable between both groups. A single stent was used in 12 cases and 2 stents in Y-configuration in 3. Thromboembolic complications occurred more often with stent assistance (33.3% vs. 8.0%, *p* = 0.002), while ischemic stroke rates were comparable between both groups (0% vs. 1.8%, *p* = 1.0). Six-month angiographic follow-up showed complete occlusion, neck remnants and aneurysm remnants in 73.4%, 19.4% and 7.3% after WEB only, respectively, and in 66.7%, 20.0% and 16.7% after WEB plus stent, respectively (*p* = 0.538). A case report shows that WEB deployment through the struts of a previously implanted standard microstent is feasible, even if a VIA 33 microcatheter is needed.

**Conclusion:**

In the present study, stent-assisted WEB embolization had a comparable safety and efficacy profile compared to treatment by WEB only. However, stent-assisted WEB embolization requires long-term anti-platelet medication, which annihilates the advantages of the WEB as a purely intrasaccular device.

Clinical Trial registration number: N/A.

## Introduction

During the past decade, intrasaccular flow-disruption with the Woven Endobridge (WEB; Sequent Medical, Aliso Viejo, CA, USA) has evolved as a proven concept for endovascular treatment of predominantly wide-necked bifurcation aneurysms. Several prospective trials demonstrated a favourable safety and efficiency profile for the WEB, in particular regarding the subset of rather complex aneurysms typically treated with the WEB [1, 21]. In a recent meta-analysis, Zhang et al. reported complete and adequate aneurysm occlusion in 53% and 80% at short-term follow-up, respectively, and a morbidity rate of 6% [25]. Moreover, comparative studies demonstrated improved angiographic results and/or a better safety profile when directly compared to traditional treatment strategies such as coiling, stent-assisted coiling and microsurgical clipping [13, 15, 16, 19].

As a purely intra-saccular device, the WEB does not require long-term anti-platelet therapy imperatively. This represents a major advantage over stent-assisted coiling, which is the standard endovascular treatment option for wide-necked aneurysms: First, leaving the parent artery unaffected may translate into a lower risk of thromboembolic complications and ischemic stroke [12, 15]. Second, omitting anti-platelet medication reduces the risk of haemorrhagic complications and increases the quality of life of patients [2]. However, in some cases of complex aneurysm geometry, or when the delivered WEB tends to protrude into the parent artery or attenuates the blood flow by remodelling of the parent artery-aneurysm-complex, additional stent implantation may be required. In this case, long-term anti-platelet medication would become mandatory as for stent-assisted coiling. Although most WEB studies mention a small portion of aneurysms treated with stent assistance, there is a shortage of studies that analyse stent-assisted WEB embolization systematically.

The objective of this multi-centre series was to compare stand-alone and stent-assisted WEB embolization in terms of aneurysm characteristics, adverse events and angiographic outcomes. Moreover, we report the case of a complex aneurysm, in which the WEB was delivered through a previously implanted microstent in a staged procedure.

## Methods

This is a retrospective, multi-centre analysis of patients treated with the WEB between January 2011 and October 2020. An ethics committee approval was not required for this retrospective study in accordance with the institutional review boards.

### Inclusion and exclusion criteria

All patients treated with a WEB for a ruptured or unruptured aneurysm were considered for inclusion. Aneurysms with a size ≥ 11 mm were excluded, since the WEB is not recommended for these aneurysms according to the manufacturer’s instructions. Furthermore, partially thrombosed aneurysms, dissecting and fusiform aneurysms were excluded. The aneurysm in the case report was not included in the systematic analysis, as the WEB was delivered through the stent and the procedure was staged.

### Procedure

All cases were discussed within an interdisciplinary neurovascular conference. The WEB was predominantly used for wide-necked bifurcation aneurysms as an alternative treatment option for stent-assisted coiling and microsurgical clipping. The primary treatment concept of all aneurysms included into this study consisted of complete aneurysm occlusion by WEB only or in combination with coiling to provide optimal aneurysm occlusion. The decision to treat with stent-assisted WEB embolization was made based on two scenarios: (1) Accepted stent-assisted WEB embolization: Aneurysms with complex anatomy, such as very broad-based aneurysms, were primarily subjected to sole WEB embolization. If WEB protrusion into the parent artery was anticipated, consecutive stent implantation was accepted. This strategy was regarded as an alternative to stent-assisted coiling, as WEB + stent was supposed to provide more durable aneurysm occlusion. (2) Unplanned stent implantation: Following a WEB only treatment concept, the implantation of a supporting stent became necessary, if the WEB tended to protrude into the parent artery and/or attenuated the blood flow.

All procedures were performed via a transfemoral approach with the patient under general anaesthesia in a biplane angiosuite (Philips, Best, the Netherlands and Siemens, Erlangen, Germany). The WEB was delivered through a dedicated VIA microcatheter (VIA 17, 21, 27 and 33; Sequent Medical, Aliso Viejo, CA, USA) in the majority of cases.

WEB sizing was performed based on aneurysm width and height measurements on two-dimensional DSA images in working projection. Implant sizes were chosen to be slightly larger than the aneurysm width and slightly smaller than the aneurysm height (+ 1/ − 1 rule) as recommended in the manufacturer’s instructions for use.

If stent assistance was required, stents were deployed through a conventional microcatheter (Headway 17 or 21, Microvention, Tustin, CA, USA or Prowler Select Plus, Raynham, MA, USA) using a triaxial guide-catheter system. The stent type was left to the discretion of the neurointerventionalists. For stent-assisted WEB embolization, two different techniques were applied: The standard approach consisted of subsequent deployment of a microstent across the aneurysm neck to support WEB position in case of WEB protrusion. A second approach is described in the case report. First, a stent was placed across the neck of a bifurcation aneurysm. Six weeks later, when partial neoendothelialization was anticipated, the aneurysm was probed with a VIA microcatheter through the stent interstices, and subsequent WEB deployment.

### Anti-aggregation therapy

Patients received acetylsalicylic acid (ASA) 100 mg/day starting 5–7 days before a planned WEB procedure. During the procedure, a bolus of heparin (5000 IU) was administered after groin puncture, followed by aliquots of 1000 IU/h. After WEB implantation, ASA monotherapy was continued for 4–6 weeks. In unruptured aneurysms with unexpected stent implantation, the patients were loaded with clopidogrel 300 mg orally, either via nasogastric tube during the procedure or per os after the intervention. A maintenance dual anti-platelet regimen of clopidogrel 75 mg for 4 months and ASA life-long was conducted, which is our hospital standard after stent implantation. Tirofiban was not administered. Clopidogrel response was not routinely assessed afterwards.

Patients with ruptured aneurysms treated with WEB only did not receive anti-platelet therapy. For ruptured aneurysms treated with stent-assistance, tirofiban (Aggrastat, Merck, West Point, PY, USA) was started promptly before stent placement and continued for 16–24 h after the procedure, followed by an overlapping loading dose of ASA (500 mg) and additionally clopidogrel (300 mg). The maintenance anti-platelet protocol after stent implantation is similar to that of unruptured aneurysms.

### Data collection

The following parameters were retrospectively collected: patient age, sex, previous aneurysm treatment, ruptured/unruptured aneurysm status, aneurysm location, WEB type, additional coiling, adverse events and retreatment. The following WEB types were used: WEB double-layer (DL), single-layer (SL) and single-layer sphere (SLS). Both the WEB 17 and its predecessor versions were used. Neurological assessment was at least performed at baseline, after the procedure and at discharge by a clinical neurologist or neurosurgeon not involved in the intervention. We report both symptomatic and technical/asymptomatic events. In this context, both haemorrhagic and thromboembolic events are subdivided into symptomatic and technical/asymptomatic complications. Neurological complications were defined to be associated with transient or permanent neurological deficits after the procedure. Ischemic stroke was denoted as neurological worsening with correlating ischemic lesions on (perfusion) computed tomography and/or magnetic resonance imaging.

### Angiographic evaluation

Conventional four-vessel digital subtraction angiography (DSA) scans were reviewed in order to determine aneurysm dome width, height and neck width. Based on these parameters, the dome-to-neck ratio and the aspect (height-to-neck) ratio were calculated [9]. The maximum aneurysm diameter is the largest value of either aneurysm width or height [8]. A wide neck was defined as a neck width ≥ 4 mm and/or a dome-to-neck ≤ 2 [9]. Aneurysm occlusion was evaluated immediately after the procedure and at 6-month angiographic follow-up, applying the Raymond-Roy occlusion classification (RROC): (1) complete occlusion, (2) neck remnant and (3) aneurysm remnant. Complete occlusion and neck remnants were subsumed as adequate occlusion.

### Statistical analysis

Qualitative parameters are presented as numbers and percentages and compared with the chi-square and the Fisher’s exact test. Quantitative parameters are presented as means with standard deviation and groups were compared with the unpaired Student’s *t*-test and the Mann–Whitney-*U*-test. Normality was evaluated with the Shapiro–Wilk-test. Factors with a *p* < 0.05 in the univariate analysis were entered into a step-wise bivariate logistic regression model to identify independent factors associated with the respective outcome measures. Statistical analysis was performed using SPSS software (IBM SPSS Statistics for Windows, Version 25.0, Armonk, NY, USA). A *p*-value < 0.05 was considered as statistically significant.

## Results

### Patient and aneurysm characteristics

A total of 178 patients met the inclusion criteria and were enrolled. The mean patient age was 58.4 ± 12.0 years and 126 (70.8%) were female. There were 54 (30.3%) ruptured aneurysms and 9 (5.1%) recurrent aneurysms. The aneurysms were located in the anterior circulation in 109 (61.2%) cases and in the posterior circulation in 69 (38.8%). The mean maximum aneurysm diameter was 7.0 ± 2.4 mm, with a mean dome width of 6.1 ± 2.2 mm and a mean aneurysm height of 6.4 ± 2.5 mm. The mean neck width was 4.3 ± 1.6 mm and 163 (91.6%) were wide-necked.

A total of 163 (91.6%) aneurysms were treated by WEB only and 15 (8.4%) were treated with stent-assistance. The baseline characteristics were not significantly different between the two groups, as outlined in Table 1.

**Table 1:** Baseline patient and aneurysm characteristics

	WEB only *(n* = 163)	WEB + stent (*n* = 15)	*P*
Patient age (years)	58.0 ± 12.3	61.8 ± 7.4	0.246
Female sex	114 (69.9%)	12 (80.0%)	0.558
Ruptured aneurysm status	51 (31.3%)	3 (20.0%)	0.558
Aneurysm location			
Acom	43 (26.4%)	4 (26.7%)	1.0
MCA	26 (16.0%)	2 (13.3%)	1.0
ICA	3 (1.8%)		
Paraophthalmic	11 (6.7%)	2 (13.3%)	0.301
Pcom	13 (8.0%)	1 (6.7%)	1.0
Terminus	4 (2.5%)	0 (0%)	1.0
Posterior circulation	63 (38.7%)	6 (40.0%)	0.918
Bifurcation location	133 (81.6%)	10 (66.7%)	0.164
Aneurysm size			
Maximum diameter (mm)	7.0 ± 2.3	7.0 ± 3.3	0.978
Aneurysm width (mm)	6.0 ± 2.0	6.6 ± 3.2	0.531
Aneurysm height (mm)	6.4 ± 2.5	5.9 ± 0.8	0.472
Neck width (mm)	4.2 ± 1.5	5.1 ± 2.6	0.211
Dome-to-neck ratio	1.5 ± 0.5	1.3 ± 0.5	0.216
Aspect ratio	1.6 ± 0.8	1.2 ± 0.4	0.059
Wide neck	148 (90.8%)	15 (100%)	0.619
Lobulated morphology	13 (8.0%)	1 (6.7%)	1.0
Recurrent aneurysm	7 (4.3%)	2 (13.3%)	0.169

*WEB*, Woven Endobridge; *Acom*, anterior communicating artery; *MCA*, middle cerebral artery; *ICA*, internal carotid artery; *Pcom*, posterior communicating artery

### Aneurysm treatment

Procedural specifics are detailed in Table 2. The WEB DL was used in 11 (6.2%) cases, the WEB SL in 133 (74.7%) and the WEB SLS in 34 (19.1%). There were no significant differences between the two groups among these WEB types. The WEB 17 required stent-assistance less frequently than its predecessor WEB systems (4.0% vs. 11.7%); however, this difference was not statistically significant (*p* = 0.1).

**Table 2:** Procedural specifics

	WEB only (*n* = 163)	WEB + stent (*n* = 15)	*P*
WEB type			
DL	10 (6.1%)	1 (6.7%)	1.0
SL	124 (76.1%)	9 (60.0%)	0.170
SLS	29 (17.8%)	5 (33.3%)	0.168
WEB 17	72 (44.2%)	3 (20.0%)	0.100
Additional coiling	4 (2.5%)	2 (16.7%)	0.082

*WEB*, Woven Endobridge; *DL*, dual-layer; *SL*, single-layer; *SLS*, single-layer sphere

In the WEB + stent group, 12 aneurysms were treated with a single stent and 3 bifurcation aneurysms were treated with 2 stents in Y-configuration. An Enterprise stent (Codman Neurovascular, Raynham, MA, USA) was used in 13 cases, a Solitaire stent (Medtronic, Irvine, CA, USA) in 3, a Neuroform Atlas (Stryker Neurovascular, Kalamazoo, MI, USA) in 1 and a LEO baby stent (Balt Extrusion, Montmorency, France) in 1.

### Complications

Procedural complications are listed in Table 3. The overall complication rate was 12.4% (22/1178), including 18 thromboembolic and 3 haemorrhagic events. Overall thromboembolic events occurred more often during stent-assisted WEB embolization (33.3%) than during WEB embolization only (8.0%, *p* = 0.002). In most cases, the thrombus could be resolved with tirofiban or acetylsalicylic acid and/or the patient had asymptomatic (silent) infarction. Hence, symptomatic thromboembolic events (ischemic stroke) were not significantly different (0% vs. 1.8%, *p* = 1.0). In the stent-assisted WEB embolization group, 3 of 5 thromboembolic events occurred due to WEB protrusion into the parent artery and 2 were related to stent-implantation.

**Table 3:** Procedure-related technical/asymptomatic and symptomatic complications

	WEB only (*n* = 163)	WEB + stent (*n* = 15)	*P*
Overall complications	16 (9.8%)	6 (40.0%)	0.001
Symptomatic complications	5 (3.1%)	1 (6.7%)	0.415
Thromboembolic complications	13 (8.0%)	5 (33.3%)	0.002
Technical/asymptomatic	10 (6.1%)	5 (33.3%)	
Symptomatic	3 (1.8%)	0 (0%)	
Haemorrhagic complications	3 (1.8%)	0 (0%)	1.0
Technical/asymptomatic	1 (0.6%)	0 (0%)	
Symptomatic	2 (1.2%)	0 (0%)	
Neurological complications	5 (3.1%)	1 (6.7%)	0.415
Ischemic stroke	3 (1.8%)	0 (0%)	1.0
Brain oedema	0	1 (6.7%)	0.084

There was one neurological complication related to stent-assisted WEB embolization (6.7%): After WEB placement in an unruptured Acom aneurysm, the WEB protruded into the parent vessel requiring additional stent implantation. After the procedure, the patient showed transient aphasia and a mild hemiparesis on the right. Emergency CT showed vasogenic oedema around the Acom aneurysm, but there was no infarction. The patient recovered fully. There was no ischemic stroke in the WEB + stent group.

There were 5 (3.1%) neurological complications in the WEB only group, including 3 ischemic strokes and 2 haemorrhagic events (1 aneurysm rupture, 1 rupture of a perforating artery). One patient with ischemic stroke and one with procedural aneurysm rupture died.

Among ruptured aneurysms, thromboembolic events occurred in 15.7% (8/51) after WEB only treatment and in 33.3% (1/3) after stent-assisted WEB embolization.

### Angiographic outcome

Angiographic results are summarized in Table 4. Immediate complete and adequate occlusion was achieved in 47.9% and in 65.0% after WEB only treatment, respectively, and in 60.0% and 80.0% after stent-assisted WEB embolization, respectively. At 6-month follow-up, complete and adequate occlusion rates were 73.4% and 92.7% in the WEB only group, respectively, and 66.7% and 86.7% in the WEB + stent group, respectively. Ten patients (5.6%) were retreated, 9 (7.3%) after WEB implantation only and 1 (6.7%) after stent-assisted WEB embolization. Angiographic outcome and retreatment rates were not significantly different between the two groups.

**Table 4:** Angiographic results

	Immediate aneurysm occlusion		6-month angiographic follow-up	
	WEB only (*n* = 163)	WEB + stent (*n* = 15)	WEB only (*n* = 124)	WEB + stent (*n* = 15)
Complete occlusion (RROC 1)	78 (47.9%)	9 (60.0%)	91 (73.4%)	10 (66.7%)
Neck remnant (RROC 2)	28 (17.2%)	3 (20.0%)	24 (19.4%)	3 (20.0%)
Aneurysm remnant (RROC 3)	57 (35.0%)	3 (20.0%)	9 (7.3%)	2 (13.3%)
Adequate occlusion	106 (65.0%)	12 (80.0%)	115 (92.7%)	13 (86.7%)

*RROC*, Raymond-Roy occlusion classification

### Case report

**Fig. 1:**
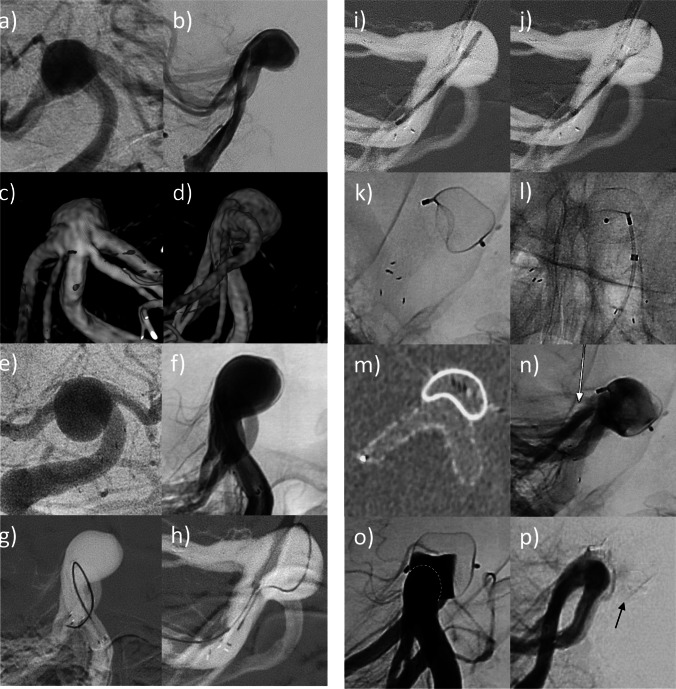
Digital subtraction angiography (**a**–**b**) with three-dimensional reconstructions of rotational angiography (**c**–**d**) shows an unruptured, wide-necked aneurysm at the basilar tip (10 × 6 mm). The neck width (10 mm) equals the aneurysm width, resulting in a dome-to-neck ratio of 1. The aneurysm is tilted anteriorly and incorporates both PCA trunks in its base. Due to its broad base, aneurysm embolization with stent-assisted coiling was planned. At first, a Neuroform Atlas microstent (4.5 × 30 mm) was implanted from the right P1 to the basilar artery (**e**–**f**). Subsequent coil deployment within the aneurysm failed, as already the first coil could not be secured in the aneurysm sac and protruded into the parent vessel, as the broad base was not fully covered by the single stent (**g**). Hence, implantation of a second stent in Y-configuration was attempted. However, probing of the left P1 through the struts of the first stent failed, although trying various microwire-microcatheter combinations (**h**). Given that the single stent covered the broad base only partially, we supposed that a WEB could be placed better within the aneurysm than the coils due to its balloon-like shape. In a staged procedure, a WEB SL 11 × 6 mm should be deployed based on the aneurysm dimensions. After successful probing of the aneurysm sac through the stent struts with the VIA 0.033-inch microcatheter (**i**), the WEB could be correctly deployed within the aneurysm sac (**j**). The previously implanted stent shouldered the WEB at its bottom and supported correct WEB position (**k**–**m**). Immediate angiographic control showed contrast stasis within the aneurysm and patency of the left P1 (**n**, arrow). Three-month follow-up showed progressive aneurysm occlusion of the aneurysm with residual perfusion of the aneurysm base; the dashed line indicates the contour of the intracranial stent (**o**). At 12-month follow-up, the aneurysm was completely occluded and the parent vessel remained patent; the arrow indicates the tip of the completely occluded WEB (**p**)

The case report is presented in Fig. 1. A 58-year-old female presented with an unruptured, wide-necked aneurysm at the basilar tip (10 × 6 mm). After interdisciplinary case discussion, endovascular treatment was recommended due to the large aneurysm size and a bleb at the apex of the aneurysm. Owing to a broad neck (10 mm) and parts of the aneurysm incorporating the basilar bifurcation, the aneurysm was supposed to be treated with stent-assisted coil embolization. At first, a Neuroform Atlas microstent (4.5 × 30 mm) was implanted from the right P1 to the basilar artery. Attempting coil embolization, the introduced coil tended to protrude into the parent vessel, so that it could not be detached and had to be removed. Subsequently, placing a second microstent into the left P1 in Y-configuration was attempted; however, probing of the left P1 through the struts of the first stent failed, although trying various microwire-microcatheter combinations. Hence, we decided to complete the final aneurysm embolization 6 weeks later, when partial neoendothelialization was anticipated in order to reduce the risk of stent dislocation. Due to failure of a Y-stenting attempt, a WEB implantation through the stent interstices was envisaged. After successful probing of the aneurysm sac through the stent interstices with a convenient VIA 33 microcatheter (diameter: 0.0033 inch), the WEB SL 11 × 6 could be correctly placed within the aneurysm sac. The previously implanted stent shouldered the WEB at its base and supported correct WEB position. Hence, complete aneurysm embolization was achieved without attenuating blood flow in the parent artery. Three-month follow-up showed progressive aneurysm occlusion of the aneurysm and complete aneurysm occlusion after 12 months. There were no procedure-related adverse events.

## Discussion

In the current study, additional stent implantation was performed in 9.4% of all WEB procedures. A wide neck and sidewall aneurysm location were independent factors associated with stent implantation. Complication rates and angiographic outcome were comparable between both treatment regimens.

The efficiency of the WEB procedure is well documented in the available literature. In the cumulative population of the three prospective studies, WEBCAST (WEB Clinical Assessment of Intrasaccular Aneurysm), WEBCAST-2 and French Observatory, complete and adequate occlusion rates were 52.9% and 79% at 1 year, respectively [21]. During long-term follow-up, complete and adequate occlusion were reported in 51.2% and 81.0% after 2 years, respectively, and in 50.8% and 83.6% after 3 years, respectively [20, 22]. Between 1- and 3-year follow-up, aneurysm occlusion was stable in 86.9% [22]. In the selected aneurysm subset of these three benchmark studies, additional stents were used in 5 of 163 aneurysms (3.1%) to ensure optimal WEB position [21]. In the prospective WEB-IT study, additional stents were required in 2 of 150 aneurysms [1].

Among retrospective studies, additional stents were implanted in 4.9% in the study by Fujimoto et al. [7], in 7.8% in the study by Popielski et al. [23] and in 19.2% in the study by Mine et al. [18]. In the current study, stent-assistance was necessary in 9.4% of WEB procedures, which is within the range of the cited studies.

To date, the study by Cagnazzo et al. is the only one that focuses on stent-assisted WEB embolization [3]. Among 102 procedures, additional stent implantation was performed in 17 patients (16.7%). The most common artery location was the MCA, followed by the Acom and the basilar tip. All aneurysms were located at a bifurcation or had a branching vessel from the neck. The authors observed 2 cases of reversible in-stent thrombosis, which remained asymptomatic. At angiographic follow-up, complete and adequate occlusion rates were 69% and 81.5%, respectively, which were comparable to the above cited benchmark studies. These findings correlate with the angiographic results obtained in our study. Furthermore, stand-alone and stent-assisted WEB embolization provided similar aneurysm occlusion after multivariable adjustment.

In the study by Cagnazzo et al., auxiliary stents were predominantly used for bifurcation aneurysms [3]. In contrast, stent-assisted WEB embolization was significantly more often performed for side-wall aneurysms in the present study, such as the paraophthalmic segment of the ICA, the Pcom and the vertebral artery. Owing to the advances in neurointerventional technique, intracranial stents are suitable for both bifurcation and side-wall aneurysms. For bifurcation aneurysms, Y-stenting in combination with coiling has gained increasing popularity, and represents an alternative endovascular treatment option for bifurcation aneurysms to WEB embolization. In the meta-analysis on Y-stent assisted coiling by Cagnazzo et al., long-term complete and near-complete aneurysm occlusion was achieved in 95.4% (95%CI: 93.7–97.0%) [4]. For comparison, mid-term adequate occlusion rates were 92.7% for WEB only and 88.2% for WEB plus stent in the present analysis. Also, the novel WEB 17 required stent-assistance less frequently than its predecessor versions; however, this difference was mitigated after multivariable adjustment and may be related to the fact that the WEB 17 only allows treatment of small aneurysms (≤ 7 mm) [11].

A special technique of stent-assisted WEB embolization was presented in the case report. The basilar tip aneurysm incorporated partially the basilar bifurcation and had a very broad neck with a dome-to-neck ratio of 1.0. Therefore, coiling or WEB embolization without stent-assistance would not be feasible, as the devices would protrude into the parent artery. In the presented case, Y-stent-assisted coiling was envisaged. After placement of the first stent, however, we faced two problems with this approach: First, probing of the left PCA with microwire and microcatheter through the stent interstices failed technically, although various microwire/microcatheter-combinations were attempted. Second, pursuing coil embolization with a single stent strategy failed, because already the first coil tended to protrude into the parent artery owing to the broad base reaching from the right to the left P1. Due to the barrel-like configuration of the WEB, we anticipated that a single stent, which covered about two-thirds of the neck, would suffice as counterfort to support correct device position within the aneurysm sac. Owing to the large aneurysm size, the largest available WEB was necessary, which can only be delivered through the largest, 0.033-inch VIA microcatheter. In the reported case, probing the aneurysm through the stent interstices with the VIA 33 microcatheter and deployment of the WEB SL 11 × 6 was unproblematic. In particular, the stent shouldered the WEB at the level of the aneurysm neck, resulting in aneurysm occlusion while maintaining undisturbed blood flow in the parent artery. Because of extended procedure time (failure of Y-stenting attempt) and to minimize the risk of stent dislocation by probing the stent interstices with the VIA 33, we decided to stage the procedure. Animal and post-mortem studies have suggested that stent neoendothelialization commences within 6 months after stent implantation [17]. Yet, in our experience and as suggested by other authors, staged aneurysm embolization 6 weeks after stent implantation is sufficient to avoid stent migration [5].

From our personal experience, reprobing the aneurysm sac through the stent struts with the microcatheter works best with laser-cut stents, since metal coverage is usually lower and porosity higher compared to their braided counterparts. Among laser-cut stents, we prefer open-cell stents such as the Neuroform stents to closed-cell stents as they have larger uncovered gaps between the struts and tend to an emphasized opening of their stent pores with less ovalization in bended anatomy [6].

Although the approach/strategy of WEB embolization through a stent was successful in this case, further validation of this technique will be necessary for different locations and aneurysm geometries to draw a definite conclusion. Nevertheless, this technique has the potential as rescue strategy, when stent-assisted coiling has failed and treatment by WEB or coil only is not possible for anatomical reasons.

Previous studies showed that stent-assisted procedures carry a higher risk for ischemic stroke caused by thromboembolism or in-stent stenosis than conventional coiling. For instance, 1-year ischemic stroke rates were 8.8% for stent-assisted coiling and 2.2% for coiling in the study by Hetts et al. [14]. As an intrasaccular device, the WEB is supposed to carry a lower risk of thromboembolic complications than stent-assisted procedures and does not require long-term anti-platelet treatment. In the comparative study by Kabbasch et al., the ischemic stroke rate of the WEB (1.5%) was lower than that of stent-assisted coiling (6.1%) [15]. The concept of stent-assisted WEB embolization would mitigate the inherent advantage of the WEB as a purely intrasaccular device, and would naturally increase the risk of thromboembolic events and require long-term anti-platelet therapy [10]. In the present study, the thromboembolic event rate of stent-assisted WEB embolization was 33%, caused by WEB protrusion into the parent artery in 60% and 40% were stent related. This rate appears comparably high; however, the WEB + stent group was small, which impedes a direct comparison, and all events were asymptomatic with no incidence of ischemic stroke. Our institutional anti-platelet protocol in cases of unexpected stent implantation in scheduled cases consists of loading with clopidogrel (300 mg) during the procedure via the nasogastric tube or per os after the procedure since mono-antiaggregation with acetylsalicylic acid is available. Zi-Liang et al. reported that intravenous administration of tirofiban during stent-assisted procedures reduces significantly overall thromboembolic events with no additional intracranial haemorrhages when compared to oral double anti-platelet therapy with clopidogrel and acetylsalicylic acid [26]. Hence, tirofiban administration may further increase the safety of WEB procedures with unexpected stent implantation in scheduled and emergency cases.

Given the limited evidence on stent-assisted WEB embolization, a definite conclusion cannot yet be drawn. In particular, it remains unclear, whether stent-assisted WEB embolization is associated with a higher safety or efficacy profile than stent-assisted coiling for a specific subset of intracranial aneurysms. Also given the increased treatment costs of WEB + stent, a comparative assessment of the two treatment strategies and prospectively a cost-effectiveness study would be required to define, whether stent-assisted WEB embolization has a true advantage over stent-assisted coiling as a first-line treatment. Of note, in the majority of cases, stent-assisted WEB embolization was not initially planned as standard procedure but the result of antagonizing WEB protrusion in order to optimize WEB positioning or to have a treatment alternative if Y-stent-assisted coiling was technically not feasible. Moreover, it has to be kept in mind that stent-assisted WEB embolization annihilates the advantages of the WEB as an intrasaccular device. However, based on the available data, stent-assisted WEB treatment appears to be reasonable safe and efficient as a rescue treatment after failed WEB only treatment or for complex aneurysms as an alternative treatment option to stent-assisted coiling.

### Limitations

The study is limited by the retrospective design and the moderate number of included patients. In particular, the number of patients treated by additional stent implantation was rather small. As stents were predominantly used as a rescue strategy after failed WEB only treatment, aneurysm characteristics were heterogenous in both groups, limiting a direct comparison. Furthermore, a comparison is hampered by a small number of patients treated by WEB and stent, the analysis of both ruptured and unruptured aneurysms and the different types of retreatments in both groups. Moreover, angiographic follow-up is not complete and long-term angiographic outcome was not reported. While some patients were lost to follow-up, others had CTA or MRA follow-up at an external institution and the images were not available in our PACS system. Finally, aneurysm occlusion was not determined by a core laboratory which might bias the interpretation of the angiographic results [24].

## Conclusions

In the current study, additional stent-implantation was performed in 9.4% of WEB procedures, especially for side-wall and wide-necked aneurysms. The results indicate that stent-assisted WEB embolization has a similar safety and efficacy profile than WEB only treatment. WEB deployment is also feasible through the stent struts of a previously implanted stent, as demonstrated for a single case, even if the largest available VIA microcatheter had to be used. Due to the limitations of a retrospective study and the paucity of available data, further studies will be necessary to confirm our findings. Moreover, stent-assisted WEB embolization requires long-term anti-platelet medication, which annihilates the advantages of the WEB as a purely intrasaccular device. To this point, stent-assisted WEB implantation can serve as a rescue treatment after failed WEB only treatment or for complex aneurysms, as an alternative treatment option to stent-assisted coiling.
